# Editorial: Orchestration of an Immune Response to Respiratory Pathogens

**DOI:** 10.3389/fimmu.2019.00690

**Published:** 2019-04-09

**Authors:** Steven M. Varga, Andrea J. Sant

**Affiliations:** ^1^Microbiology and Immunology, University of Iowa, Iowa City, IA, United States; ^2^Microbiology and Immunology, David H. Smith Center for Vaccine Biology and Immunology, Rochester, NY, United States

**Keywords:** influenza, T cells RSV, Tuberculosis, CD4 T cells, CD8 T cells

This issue of Frontiers deals with the complex series of events and long-term consequences of immune responses to respiratory pathogens. In this issue, the contributors discuss the earliest events following infection, the alternative paths that the adaptive immune response can take and long-term immunity that becomes established, both locally and systemically. The diversity of respiratory pathogens that present challenges to immune protection in humans is substantial and, as discussed in this issue, includes a large number of distinct respiratory viruses, including respiratory syncytial virus, metapneumovirus, rhinovirus and influenza virus (Ağaç et al.; Ascough et al.; Devarajan et al.; Fonseca et al.; Ivanciuc et al.; Richards et al.; Schmidt and Varga) as well as bacterial pathogens such as tuberculosis (Choreño Parra et al.; Orme and Henao-Tamayo). These human pathogens differ in their tropism, primary site of infection within the respiratory tract, degree of pathogenicity, seasonality and potential for chronic infection. Here, we summarize some of the key concepts that are discussed in this issue and cite recent reviews by others to provide additional background.

A consistent theme discussed by several contributors (Ascough et al.;  Schmidt and Varga; Ivanciuc et al.) is the characteristic nature that the earliest innate and inflammatory response initiates upon respiratory infection with alternate pathogens (1–6). Particularly striking in this regard is the contrast in the elicited response between RSV and influenza virus, a topic considered in detail by Ascough et al. RSV and influenza viruses differ in their primary tropism within the respiratory tract, as well as the source and abundance of early pro-inflammatory cytokines, such as type I IFNs. These elements, together with TLR-mediated differences, lead to distinctive patterns in the subsequent adaptive immune response. Typical seasonal influenza virus is characterized by dominant and protective Th1 response, while RSV is dominated by Th2/Th17 responses. As discussed by Fonseca et al., the impact of diminished type I IFN is particularly exacerbated in infants infected with RSV, who also exhibit delayed IL-12 production and IFN-γ, relative to cytokines such as IL-6 and IL-23. Together, these early soluble mediators are associated with lung immunopathology rather than protective immediate and long-term immunity. This early pattern of CD4 T cell responses to RSV is also associated with fewer polyfunctional CD8 T cells and weak protective antibody responses. Together, this pattern of immune responses to RSV poorly protects the host from future infection with even the same strain of RSV and biases the character of subsequent responses throughout life (Ascough et al.; Ivanciuc et al.). In contrast, the highly diverse population of responding CD4 T cells to influenza infection exhibits robust IFN-γ production in the lung-draining lymph node and the cohort of influenza-specific CD4 T cells that leaves the lymph node and localizes to the lung tissue becomes even more enriched in the Th1-IFN-γ dominated phenotype (Richards et al.).

A key feature in understanding and ultimately manipulating T cell immunity to respiratory pathogens requires insight into the factors and mediators that contribute to homing, extravasation, and establishment of tissue residence. Many of the contributors to this volume address this issue and pointed out the significant areas of uncertainty in these fate decisions (Reagin and Klonowski; Souquette and Thomas; Topham and Reilly). Particularly for CD4 T cells, the effector function and fate after priming is heterogeneous (5, 7), and includes follicular helper cells, that remain in the lymph node and facilitate B cell responses, prototypical Th1 cells either enter recirculation or home to the lung and cytotoxic CD4 T cells that are primarily detected in the respiratory tract. The elements within the lung draining lymph node that control commitment to these alternate fates are not well understood, but as discussed by Richards et al., it is clear that the repertoire of T cells that migrate to the respiratory tract are extremely broad in antigen specificity and in T cell receptor diversity, allowing for significant breadth in epitope recognition in the infected tissue. As discussed by Walling and Kim, integrins, such as LFA-1 and its ligand ICAM-1, play a key role in the multiplicity of T cell events involved in priming, extravasation, and delivery of effector functions in the respiratory tract. Chemokine receptors such as CXCR3, CXCR4, and CCR5 (Reagin and Klonowski;  Richards et al.; Topham and Reilly) on T cells and on other effector cells, such as NK cells (Ascough et al.; Carlin et al.), control recruitment and positioning of lymphoid cells to lung in response to ligands produced within the respiratory tract (e.g., CXCR3 with its ligands CXCL9/CXCL10 and CCR5 with its ligands CCL3/CCL5/CCL4).

As discussed by Topham and Reilly, within the respiratory tract, an extensive degree of lymphocyte compartmentalization takes place, which we are only beginning to fully understand. Evidence to date suggests that differences in localization between CD4 and CD8 T cells exists, as well as among subsets of CD8 T cells. These issues are discussed in the context of the receptor ligand pairs between T cells and tissue, such as CD103-E-cadherin, VLA-1–collagen IV, and CD49b-Collagen I that contribute to these distinct patterns of localization. Within the lung, the expression of even prototypical markers of “resident” cells, such as CD69 and CD103 are heterogeneous, again suggesting microheterogeneity in the fate and localization of the effector cells that migrate to the lung and establish residency. The expression of these receptors on T cells that control localization, retention, and even survival of T cells within the tissue are in turn regulated by complex events at priming but also by cytokines within the tissue, such as TGF-β and chemokines such as CCL25 (Topham and Reilly).

The effector functions delivered to the respiratory tract can contribute profoundly to viral clearance and resistance to future infection (Devarajan et al; Richards et al.; Schmidt and Varga;  Souquette and Thomas; Topham and Reilly). Schmidt and Varga in particular, consider the types of approaches that have been used to identify the role of CD8 T cells in viral clearance and the diversity of effector mediators in the lung. Molecules such as Fas, TRAIL, and granzyme have been shown to mediate direct contact-mediated killing of infected cells within the respiratory tract and to be mediators in ultimate viral clearance. As discussed (Schmidt and Varga), identification of key mediators involved in these functions, such as killing of infected cells, recruitment of innate effectors and production of IFN-γ have largely been derived from animal studies where genetic approaches can be used to eliminate candidate mediators and cell types. However, in humans, although many of the same mediators of protection are expressed in effector CD8 or CD4 T cells, their role in protection can only be inferred by correlative studies. There have been a limited number of such studies implicating cytotoxicity and IFN-γ in some human studies of infection. Human challenge models, although costly and labor intensive, are the most rigorous way to identify correlates of protection and sensitivity to infection for pathogenic organisms and for evaluating vaccine candidates (8–10). It is also important to appreciate that the factors that mediate protection, such as the cytotoxic mediator Fas and the cytokines IFN-γ and TNF also can contribute to immunopathology. The events that control T cell mediated immunopathology vs. protection in humans are poorly defined presently (11, 12). For the design and administration of future vaccine candidates, it is critical to gain further insight into these factors.

Many of the contributors discussed the long standing evidence for the role of memory CD8 T cells and CD4 T cells in protection from repeat infections by respiratory viruses (Ascough et al.; Devarajan et al.; Schmidt and Varga; Topham and Reilly) and tuberculosis (Orme and Henao-Tamayo), providing both homologous and heterologous protection (Souquette and Thomas). Choreno Parra et al. also present the increasing evidence of memory in natural killer cells and other innate cells in viral infection and tuberculosis. Priming via infection elicits distinct subsets of cells that persist well after pathogen clearance. As discussed by many of the articles in this issue (Choreño Parra et al.; Orme and Henao-Tamayo; Schmidt and Varga; Topham and Reilly), for the most part, these subsets are defined by the array of cell surface proteins expressed. Protective tissue resident memory CD4 and CD8 T cells have been widely implicated as important for long term protection from many pathogens (11, 13–20). However, there is evidence, discussed by Reagin and Klonowski, that within the lung, resident T cells, defined by the expression of CD69 and CD103, wane over a period of weeks to months, in both the airways and in the tissue. These authors also suggest that the lung is inherently immunosuppressive, and populated by regulatory T cells and cells that produce suppressive cytokines and that express molecules such as PD-1 ligands. Also playing a role in the diminished persistence of tissue resident cells in the lung is diminished expression of cytokines such as TGF-β that promotes expression of CD69 and CD103, the molecules that prevent egress from the tissue and promote adherence of T cells to epithelial surfaces, respectively. These authors suggest that the ultimate loss of tissue resident cells is necessary to prevent immunopathology. Clearly, in consideration of this issue, it is important to assess the normal inflammatory state of the human respiratory tract during its repeated exposure to respiratory pathogens (21). Also critical in assessing this issue is development of improved methods to quantify long term T cell memory in the respiratory tract, using such methods as *in situ* imaging that do not rely on successful extraction or expression of particular surface markers (22, 23). Ultimately, it will be important to explore the costs vs. benefits of generating and sustaining resident T cell memory for long-term protection from lung pathogens such as influenza virus and tuberculosis in future vaccine strategies that seek to amplify lung-specific immunity.

Beyond the important insights and paradigms that are derived from the detailed studies in animals, there are additional points to consider in understanding, predicting, and manipulating protective immunity to respiratory pathogens in humans. First, the primary response to most respiratory infections, generally used in animal models of infection and vaccination, only occurs once in the very long life span of humans. The vast majority of respiratory pathogens infect repeatedly, typically seasonally, in the human host. Reinfection sometimes occurs with a genetically identical strain, such as with RSV and in other cases, with a variant strain, the most typical pattern of influenza virus. Therefore, in humans, the secondary response and all of the subsequent responses will be contributed in whole or in part by memory cells which will compete with or influence the subsequent response. Beyond recruitment of lymphoid cells specific for the same pathogen, the long life span and the diversity of infections suffered by the human host (21, 24), an additional factor that is likely to play a substantial role in human immunity to respiratory pathogens is the considerable cross reactivity that exists in the T cell repertoire. This antigen/epitope cross reactivity leads to elicitation of heterologous immunity (25). Cross-reactive immunity, occurring in both chronic and acute infections to unrelated pathogens, is considered in detail by Souquette and Thomas. The cross-reactive response perturbs the trajectory of the responses that characterize the primary response in animal models of infection and that are observed in infancy and early childhood. Even with our limited understanding of this complexity, it is apparent from some examples in both animal and human studies, that such effects of heterologous immunity, induced by pathogens or vaccines, can both enhance protection or exacerbate the pathological effects of subsequent infection. Heterologous immunity can involve either immunity specific for chronic pathogens such as EBV and CMV or that specific for acutely infecting pathogens such as RSV and influenza and such cross reactivity can modulate both the effector phenotype and the responding T cell receptor repertoire. A second factor to consider in understanding human immunity to respiratory pathogens is that the respiratory tract in humans may rarely exist in the quiescent state observed in animal models maintained in specific pathogen-free facilities. A number of recent studies have documented the abundance of tissue resident memory T cells in human lung that have diverse antigen specificity (26–28). It is likely that the sustained accumulation of T cells, with a core signature of gene expression (29–31) that endow these cells with the capacity to rapidly respond to invading pathogens reflects, at least in part, the consequences of repeated and periodic confrontation of humans with respiratory pathogens. The consequences of sustaining a diverse memory pool within the lung may have pathological as well as protective consequences to the host (25, 26), depending on the individual's genetic profile, immune history, and the existence of co-morbidities. Finally, with respect to interpretation of the studies discussed in this Frontiers Research Topic, Orchestration of an Immune Response to Respiratory Pathogens and in consideration of novel vaccine strategies, it important to emphasize that the presence and function of memory lymphoid cells, whether from the innate or adaptive immune system, should not be estimated solely on the basis of complete resistance to infection. While such sterilizing immunity is the most profound and easy to assess, the contribution of memory cells to protection from pathogenic organisms can be reflected in blunted replication rates, more rapid clearance, less pathogenic disease courses as well as shorter and less efficient transmission. It is important that in the future, these parameters are monitored as effectively as possible in humans and animal models of infection and that the design and evaluation of vaccine strategies consider these benefits to the host and for preventing the spread of respiratory pathogens.

## Author Contributions

AS and SV jointly recruited authors and scope for this issue. AS reviewed submissions and relevant background literature to formulate the forward to this issue.

### Conflict of Interest Statement

The authors declare that the research was conducted in the absence of any commercial or financial relationships that could be construed as a potential conflict of interest.
